# Decreased Salinity Offsets the Stimulation of Elevated pCO_2_ on Photosynthesis and Synergistically Inhibits the Growth of Juvenile Sporophyte of *Saccharina japonica* (Laminariaceae, Phaeophyta)

**DOI:** 10.3390/plants11212978

**Published:** 2022-11-04

**Authors:** Wenze Zhang, Yunyun Shi, Lianghua He, Xinhua Chen, Fengxiao Hu, Yinrong Chen, Yun Pang, Sufang Li, Yaoyao Chu

**Affiliations:** 1 College of Marine Sciences, Fujian Agriculture and Forestry University, Fuzhou 350002, China; 2 Department of Aquaculture and Aquatic Sciences, Kunsan National University, Gunsan 54150, Korea; 3 Key Laboratory of Marine Biotechnology of Fujian Province, Institute of Oceanology, Fujian Agriculture and Forestry University, Fuzhou 350002, China; 4 Laboratoire Génie des Procédés et Matériaux (LGPM), CentraleSupélec, Université Paris-Saclay, 91190 Gif-sur-Yvette, France

**Keywords:** low salinity, ocean acidification, *Saccharina japonica*, growth, physiological traits

## Abstract

The combined effect of elevated pCO_2_ (Partial Pressure of Carbon Dioxide) and decreased salinity, which is mainly caused by freshwater input, on the growth and physiological traits of algae has been poorly assessed. In order to investigate their individual and interactive effects on the development of commercially farmed algae, the juvenile sporophytes of *Saccharina japonica* were cultivated under different levels of salinity (30, 25 and 20 psu) and pCO_2_ (400 and 1000 µatm). Individually, decreased salinity significantly reduced the growth rate and pigments of *S. japonica*, indicating that the alga was low-salinity stressed. The maximum quantum yield, F_v_/F_m_, declined at low salinities independent of pCO_2_, suggesting that the hyposalinity showed the main effect. Unexpectedly, the higher pCO_2_ enhanced the maximum relative electron transport rate (rETR_max_) but decreased the growth rate, pigments and soluble carbohydrates contents. This implies a decoupling between the photosynthesis and growth of this alga, which may be linked to an energetic reallocation among the different metabolic processes. Interactively and previously untested, the decreased salinity offset the improvement of rETR_max_ and aggravated the declines of growth rate and pigment content caused by the elevated pCO_2_. These behaviors could be associated with the additionally decreased pH that was induced by the low salinity. Our data, therefore, unveils that the decreased salinity may increase the risks of future CO_2_-induced ocean acidification on the production of *S. japonica*.

## 1. Introduction

By the end of this century, the rising atmospheric CO_2_ concentration is predicted to be within the range of 800–1000 ppm [1,2]. The increase is largely due to the use of fossil fuels and industrial activities. The ocean will dissolve more CO_2_ and reduce the pH of seawater, which is well known as Ocean Acidification (OA) [2]. Due to the significant influences of altered carbonate chemistry on biochemical cycling and marine organisms, CO_2_-induced OA has received considerable attention [3,4,5].

As primary producers, macroalgae play important roles in maintaining the biodiversity and stability of the ocean ecosystem since they provide food, habitats and nurseries for other marine organisms [6,7,8]. However, numerous studies have pointed out that CO_2_-induced OA affects algal growth, physiological properties, biochemical compositions, and even their reproductive patterns [9,10,11]. Additionally, the responses of algae to OA are species-specific [12,13]. For instance, the increased pCO_2_ (Partial Pressure of Carbon Dioxide) has been reported to stimulate the growth of *Sargassum thunbergii* [14], *Sargassum muticum* [14], *Laminaria hyperborea* [15], and *Ulva fasciata* [16]. Their greater photosynthetic rates, which are primarily favored by rising inorganic carbon, were hypothesized to be the reason for the growth enhancement. However, Gao et al. [17] and Zou [18] also noted that an elevated pCO_2_ accelerated the algal growth, but it had a small or no effect on the photosynthetic traits of *Pyropia yezoensis* and *Hizikia fusiforme*. Further, the negative impacts of elevated pCO_2_ on the biomass accumulations and photosynthetic statuses of *Saccharina japonica* [9] and *Prionitis cornea* [12] were also reported.

Apart from pCO_2_, salinity is another environmental factor that is frequently of concern [19]. Indeed, both hyper- and hyposalinity can affect the development and physiological activities of algae [20]. Particularly, the decreased salinity, which often occurs in coastal areas due to sea ice melting, rainfall and river/estuary discharge has been found to impact the growth, photosynthesis, generation span, and reproduction of seaweeds [21,22,23]. When the salinity dropped from 34 to 10 psu, the PSII photochemical efficiency of *S. japonica* was noticeably lowered [24]. Under the hyposaline condition, *Ulva linza* lengthened its generation span but distinctly reduced the pigment content and the nitrate reductase activity [22]. Li et al. [25] also demonstrated that at 5 psu, the growth, photosynthetic rate and soluble carbohydrate accumulation of *Ulva prolifera* were significantly inhibited. These responses to decreased salinity are likely associated with the changes in the intercellular ionic (e.g., Na^+^, K^+^, Cl^−^) concentrations/osmotic stress [25,26]. Due to the fact that salinity alters the solubility of CO_2_ and the carbonate equilibria of seawater [22,27], understanding how CO_2_-induced OA affects macroalgae while experiencing decreased salinity is vitally demanded.

Due to its high ecological and economic values, the brown alga *S. japonica* has become the most important component of nearshore coastal farms, particularly on the northwest coast of Pacific ocean [28]. The alga has a large market demand and has been widely used for food, fertilizer, and as raw industrial materials [29,30]. The eco-physiological or tolerance-related responses of *S. japonica* to the adverse environmental cues, e.g., elevated pCO_2_ [9,11] and high light and temperature [31,32], have been intensively reported. However, how the kelp responds to the combined OA and hyposaline conditions remains unknown. Therefore, in this work, the interplay of elevated pCO_2_ and decreased salinity on the growth and physiological traits of juvenile *S. japonica* in a short term (4 days) was assessed. To do that, the relative growth rates, pigments contents, photosynthetic parameters, and soluble carbohydrates (SC) contents of *S. japonica* that were exposed to different pCO_2_ and salinity combinations were measured. The findings are expected to shed light on how future CO_2_-induced OA coupled with low salinity affects the cultivation of commercial algae.

## 2. Results

### 2.1. Carbonate Parameters

The carbonate parameters of seawater under different pCO_2_ and salinity levels are presented in Table 1. Salinity and pCO_2_ both individually affected the seawater HCO_3_^−^ and DIC (Dissolved Inorganic Carbon) concentrations (*p* < 0.001, two-way ANOVA), and interactively impacted the CO_3_^2−^ (*p* < 0.001), CO_2_ (aq) content and pH (*p* < 0.05). For the total alkalinity (TA), only salinity had a significant effect (*p* < 0.001). The Tukey’s post hoc test depicted that except for the increasing CO_2_ concentration, the other parameters remarkably reduced with the decreasing salinity (*p* < 0.001) at two pCO_2_ levels. The elevated pCO_2_ increased the HCO_3_^−^, CO_2_ and DIC and markedly lowered the pH and CO_3_^2−^ (*p* < 0.001), but it had no effect on TA (*p* > 0.05).

### 2.2. Isolated and Interactive Effects of pCO_2_ and Salinity on Growth and Physiological Traits of S. japonica

#### 2.2.1. Relative Growth Rate

**Figure 1:**
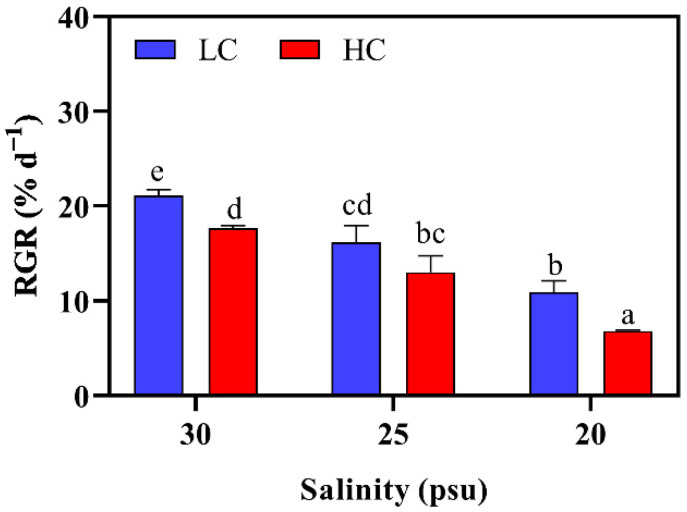
Relative growth rates of *S. japonica* grown under different pCO_2_ levels and salinities. All of the results are shown as mean value ± SD (*n* = 3). Different letters indicate significant differences (*p* < 0.05) using Tukey’s post hoc test.

The RGRs (Relative Growth Rates) of *S. japonica* under different combined conditions are shown in Figure 1. They ranged from 6.82 to 21.10 d^−1^ and were individually and drastically affected by the pCO_2_ and salinity (*p* < 0.001, two-way ANOVA, Table S1_supplementary data). The highest η^2^ (η^2^ = 0.988, Table S1_supplementary data) indicated the highest contribution of the salinity effect to RGRs. Regardless of pCO_2_ level, the RGR declined with the decreasing salinity. At the lowest salinity, the kelp that was exposed to ambient (LC) and elevated pCO_2_ (HC) levels showed reductions of 48.3% and 61.4%, respectively (Figure 1, *p* < 0.05). The elevated CO_2_ was found to further inhibit the algal growth. From 30 to 20 psu, the RGRs under HC were, respectively, 16%, 20% and 38% lower than those under the LC conditions (*p* < 0.05). No significantly interactive effects of pCO_2_ and salinity were found according to the two-way ANOVA (*p* > 0.05, Table S1_supplementary data).

#### 2.2.2. Pigment Contents

**Figure 2:**
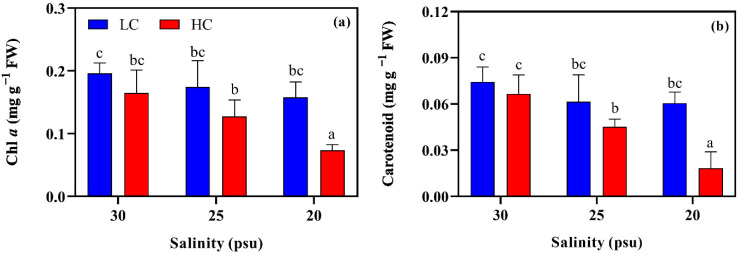
Chlorophyll *a* (**a**) and carotenoid contents (**b**) of *S. japonica* grown under different pCO_2_ levels and salinities. All of the results are shown as mean value ± SD (*n* = 3). Different letters indicate significant differences (*p* < 0.05) using Tukey’s post hoc test.

*S. japonica* showed a similar variation pattern in the Chl *a* and carotenoid contents under the tested conditions (Figure 2). Compared to the interaction between them, both the salinity and pCO_2_ individually exhibited higher influencing contributions (η^2^ = 0.57~0.66, two-way ANOVA, *p* < 0.05; Table S1_supplementary data). Under the LC condition, the pigments content slightly, but non-significantly, declined with the decreasing salinity (*p* > 0.05). However, they were further and remarkably lessened under the HC condition (*p* < 0.05). From the natural to the lowest salinity (here 20 psu), the Chl *a* and carotenoid contents, respectively, decreased from 0.16 to 0.07 mg g^−1^ FW and from 0.07 to 0.02 mg g^−1^ FW. In addition, the HC condition was also found to decrease the Chl *a* and carotenoid contents, in particular, for the thalli that were exposed to 20 psu (0.55% and 75%, respectively; *p* < 0.05, Figure 2).

#### 2.2.3. Photosynthetic Performance

**Figure 3:**
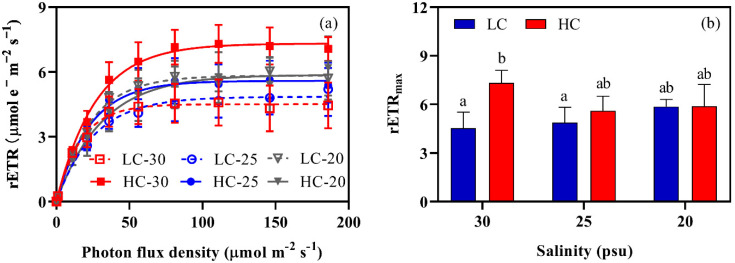
Relative electron transport rate (rETR), (**a**) and maximum fitting value of rETR (**b**) of *S. japonica* grown under different pCO_2_ levels and salinities. All of the results are shown as mean value ± SD (*n* = 6). Different letters indicate significant differences (*p* < 0.05) using Tukey’s post hoc test.

Elevated pCO_2_ and decreased salinity positively improved the RLC (Rapid Light Curves) of *S. japonica* (Figure 3a). The thalli that were exposed to the ambient pCO_2_ and salinity (LC-30) had a bottom RLC. These were utterly different from the variations for the growth rate. As displayed in Figure 3b, the interaction of pCO_2_ and salinity, and pCO_2_ alone affected the maximum electron transport rate (rETR_max_, *p* < 0.01, Table S1_supplementary data). Specifically, rETR_max_ increased under the HC condition, and the maximum value was observed at HC-30 with a value of 7.33 µmol e^−^ m^−2^ s^−1^. When it was compared to LC-30, the low salinity slightly increased the rETR_max_ (*p* > 0.05). Under the HC conditions, however, the low salinity modestly decreased the rETR_max_ (*p* > 0.05).

**Figure 4:**
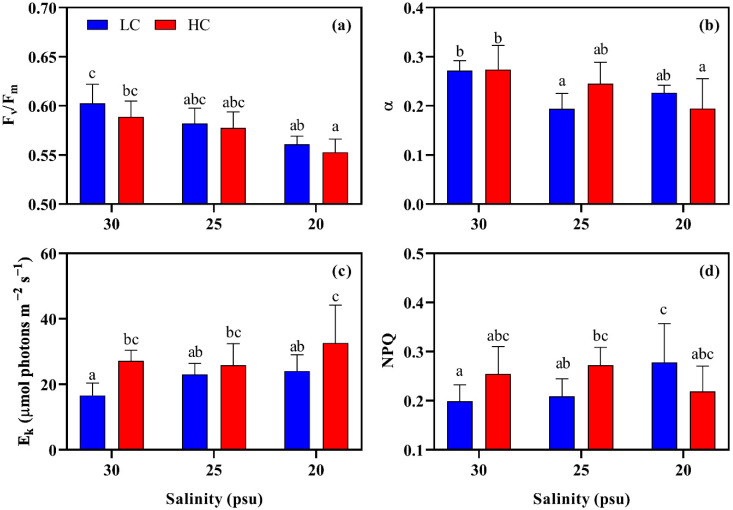
Photosynthetic parameters ((**a**) F_v_/F_m_; (**b**) NPQ; (**c**) α; (**d**) E_k_) of *S. japonica* grown under different pCO_2_ levels and salinities. All of the results are shown as mean value ± SD (*n* = 6). Different letters indicate significant differences (*p* < 0.05) using Tukey’s post hoc test.

Conversely, the maximum photosynthetic efficiency (F_v_/F_m_) of *S. japonica* was only impacted by the salinity (two-way ANOVA, η^2^ = 0.573, *p* < 0.001; Table S1_supplementary data), and it gradually reduced with the decreasing salinity (Figure 4a). At both of the pCO_2_ levels, F_v_/F_m_ significantly decreased from 0.60 to 0.55. The initial slope of RLC (α) was markedly affected by the salinity (*p* < 0.01) and the interaction between them (*p* < 0.05). As shown in Figure 4b, the lower salinities reduced α, and the minimum value (~0.19) was found in both the LC-25 and HC-20 combinations. The two-way ANOVA analysis also indicated the combined effect of pCO_2_ and salinity on the photo-saturation irradiance (E_k_, *p* < 0.05) and non-photochemical quenching (NPQ, *p* < 0.05; Table 1). Compared to LC-30, both the E_k_ and NPQ under the other combined conditions increased. For E_k_, however, only the thalli that were exposed to the HC condition presented a remarkable enhancement (*p* < 0.05, Figure 4c), especially for that at HC-20 (2.0 times). Furthermore, the salinity had no significant effect on E_k_ (*p* > 0.05). Both the elevated pCO_2_ and decreased salinities increased the NPQ, but only under the HC-25 and LC-20 combinations, while the NPQ was significantly higher (~1.40 times) than that at LC-30 (*p* < 0.05, Figure 4d).

#### 2.2.4. Soluble Carbohydrates Content

**Figure 5:**
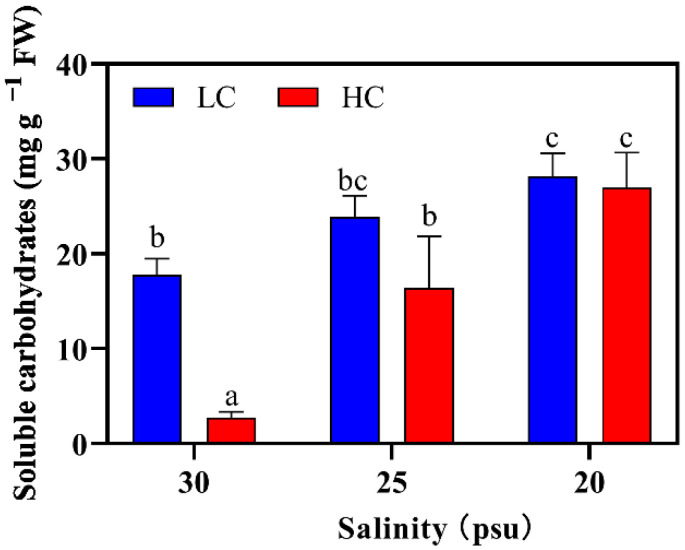
Soluble carbohydrates content of *S. japonica* grown under different pCO_2_ levels and salinities. All of the results are shown as mean value ± SD (*n* = 3). Different letters indicate significant differences (*p* < 0.05) using Tukey’s post hoc test.

The SC content was significantly affected by the salinity, pCO_2_ and their interplay (η^2^ = 0.56~0.89, two-way ANOVA, *p* < 0.01; Table S1_supplementary data). Irrespective of pCO_2_, the SC content strikingly increased with the decreasing salinity (*p* < 0.001, Figure 5). At 20 psu, the SC reached the highest content of 26~30 mg g^−1^ FW, respectively showing 1.58- and 9.84-times increases compared to those under the LC and HC condition at 30 psu. The SC content was also significantly influenced by pCO_2_. Under the HC condition, drastic (84.6%, *p* < 0.001) and slight decreases (31.2%, *p* < 0.05; Figure 5) were, respectively, observed at 30 and 25 psu. However, this reduction was finally mitigated by the decreased salinity.

## 3. Discussion

The separate effects and the interaction of salinity or pCO_2_ with the other parameters such as light intensity, temperature and nutrient availability on the growth and physiological properties of macroalgae have been extensively discussed [9,11,26,33,34,35]. However, the possible interaction between elevated pCO_2_ and decreased salinity has been poorly investigated [22,27]. In the current study, the decreased salinity and the elevated pCO_2_, individually and/or interactively, were found to significantly affect the growth, pigments contents, photosynthetic parameters, and cellular compounds of the juvenile sporophytes of *S. japonica* (Table S1_supplementary data).

Our data show that decreased salinities and elevated pCO_2_ decreased the growth rate of *S. japonica* (Figure 1), indicating that this alga was stressed and had limited resistance to the tested factors. F_v_/F_m_ is widely used as an indicator showing the “health status” of algae [36]. In this work, the F_v_/F_m_ significantly reduced with the decreasing salinity, but it did not differ between the two pCO_2_ levels at each salinity (Figure 4). Other parameters, such as growth rate, pigments and SC contents, were also mainly affected by the low salinity as depicted in the two-way ANOVA analysis (Table S1_supplementary data). These may suggest that juvenile *S. japonica* is more sensitive to the low salinity than it is to an elevated pCO_2_ condition.

The decreased salinity significantly reduced the growth rate of the juvenile *S. japonica* (by 48.3–61.4% at 20 psu, Figure 1), which could result from an energetic reallocation when the alga was coping with the low-salinity stress. Indeed, in order to maintain ionic homeostasis, a rapid influx of water and loss of ions (mainly Na^+^, K^+^, and Cl^−^) may occur in the thalli that are subjected to hyposalinity [26,37,38,39]. However, the ions’ transport is an energetic consumption bioprocess [26]. The rapid transport of water/ions through the membrane may also induce reactive oxygen species (ROS), which interfere with normal physiological metabolisms [39,40,41,42]. To minimize the ROS-induced damages, the macroalgae have been shown to upregulate antioxidant defense mechanisms, such as biosynthesizing antioxidant substrates and increasing the activities of antioxidant enzymes [25,41,43]. Thus, more energy is likely used for maintaining the osmotic equilibrium and antioxidant defense systems rather than the tissue growth. As demonstrated by Wang et al. [39], the declined growth of *Pyropia haitanensis* may be due to the redirection of energy from the growth-related processes to the hyposalinity-stress protection and survival processes.

Our previous works have shown that elevated pCO_2_ negatively impacts the growth and quality of adult *S. japonica* [9,44]. However, the knowledge on how *S. japonica* responds to elevated pCO_2_ at its early stage is also needed. The elevated pCO_2_ increased the rETR_max_ (Figure 3) and E_k_ (Figure 4), indicating that the higher pCO_2_ improved the photosynthetic capacity of the young thallus. This stimulating effect may be primarily due to the increased DIC availabilities [13,25,45,46].

Photosynthesis provides energy and carbon sources for the growth of photo-organisms. Thus, one may expect that algal growth is positively relevant to the photosynthetic rate [14,46]. However, contrary to the expectation, *S. japonica* had a diminished growth rate (16–38%, Figure 1) at the higher CO_2_ concentration, which may suggest a decoupling between the growth and the photosynthesis of this kelp. The decreased pH (Table 1) may be a reason for the detrimental effect that the elevated pCO_2_ had on growth. It is because a low pH may induce the ROS that damage cell membranes and photosystems, and inhibit the activity of enzymes that are involved in intracellular metabolisms, such as the carbon assimilation [47,48]. On the other hand, maintaining the internal acid–base balance may also require extra energy (i.e., ATP) that is derived from the decomposition of organic matters [25,48,49]. This is in line with the decreased SC content under the HC condition (Figure 5). Therefore, it is likely that in the current work, less carbon/energy was transferred to the biomass, and consequently, this reduced the growth rate. This is in accordance with the findings in other brown algae, such as *Desmarestia aculeate* and *Alaria esculenta* [50,51], as well as crustose coralline algae [46].

In brown algae, the pigments, Chl *a* and carotenoid are essential to photosynthesis as they are responsible for light-energy absorption [52]. The higher pCO_2_ and decreased salinity reduced the content of the pigments (Figure 2), suggesting that the kelp captured fewer photons for photosynthesis, and consequently, this decreased the total energy input for the growth and metabolic processes [43,53]. The alga was also found to downregulate the light utilization efficiency, as shown by the compromised F_v_/F_m_ and the initial slope (α) of RLC (Figure 4). These reductions seem to be protective strategies that avoid the photo-damage of excess energy to the photosystems and improve the algal capabilities to resist or acclimate to the adverse factors [48,54,55]. The elevated pCO_2_ decreased the pigments, and the reduction was aggregated by the low salinities. This may suggest that the decreased salinity magnified the negative effect of the elevated pCO_2_. Indeed, the decreased salinity significantly altered the bicarbonate chemicals of seawater, which especially further reduced the pH (0.12 and 0.14 unites for LC and HC, respectively). Therefore, it is not surprising that the maximum reduction in the pigments was observed in HC-20. The pH declines that are caused by the reduced salinities have been recorded in previous studies [22,27]. However, in this study, it is the first time that the negatively additive stress of an elevated pCO_2_ to the kelp in association with the decreased salinity was noted.

Although the content of pigments was downregulated, the alga under the lower salinities (including those at HC) presented a slight but non-significant increase in the rETR when they are compared to that at LC-30 (Figure 3). One may speculate that the tested factors only interfered the light-harvesting apparatuses (pigment–protein complexes), but they did not disturb the functional integrity of the photosynthetic components that are mainly involved in the electron transport events [52]. A similar regulatory pattern was shown in microalgae *Phaeodactylum tricornutum* when it coped with the stress that was caused by salicylic acid [56]. Apart from the protective strategies mentioned above (decreasing light absorption and use efficiency), the enhancement of the NPQ implicated a higher dissipation of excess energy in *S. japonica*. This procedure could also reduce the damages to the reaction centers of photosystems, where the initial charge separations and main electron transport events occur [11,57,58]. On the other hand, lower salinities slightly decreased the algal photosynthetic rate (rETR_max_, Figure 3) when they were compared to those at HC-30. This could be because the lower pH due to the decreased salinity may offset the stimulating effect that is induced by the elevated pCO_2_.

The elevated pCO_2_ enhanced the photosynthetic rate but decreased the SC content (Figure 5). The unexpected reduction may be linked to the carbohydrate decomposition, from which cells gain energy to maintain the acid–base equilibrium [48,49]. The SC was also proposed to provide C-skeletons for the biosynthesis of proteins, which probably work as antioxidant enzymes, and/or lipids when the thalli cope with the elevated pCO_2_ stress [59,60]. Likewise, the reduction of the SC due to excretion could not also be ruled out [61,62]. Such a decrease in the SC under the elevated pCO_2_ was also reported in the adult *S. japonica* when they were exposed to a nutrient-enriched condition [9]. Generally, to maintain the turgor pressure under a hyposaline condition, the cells are prone to decrease the low molecular weight carbohydrate (LMWC) content to avoid water influx [26,37,63]. In contrast, in the present study, the decreased salinity favored the SC accumulation and even mitigated the negative effect of the elevated pCO_2_. It seems that the SC did not participate in osmotic regulation, and the enhancement of it at a hyposaline condition may be interpreted as the expense of the energy for growth (Figure 1). A similar result has also been observed in the macroalgae *Ulva pertusa* [64] and microalgae *Nitzschia frustulum* [65].

Our results provide knowledge that under a future scenario of ocean acidification, low salinity that are caused by freshwater input may improve the risk of OA for the production of *S. japonica*. Individually and/or interactively, the decreased salinity and elevated pCO_2_ altered the growth, photosynthetic and biochemical profiles of the juvenile sporophytes of *S. japonica*. To further understand how these two factors synergistically affect the production of *S. japonica*, field experiments in the areas of coastal farms are also needed.

## 4. Materials and Methods

### 4.1. Samples Collection and Maintenance

The juvenile sporophytes of *S. japonica* were collected from cultivation farms that are located in Lianjiang, Fujian, China (26°07′ N, 120°17′ E) in December 2021. The samples with seedling curtains were maintained in foam boxes with cold seawater and quickly transported to the laboratory within several hours. The healthy thalli were selected and rinsed several times with sterilized seawater to remove the epiphytic organisms and detritus. Approximately 300 juvenile sporophytes (~6 cm in average length) were stock-cultured in an aerated tank containing 6 L filtered seawater (25 µM N-NO_3_^−^ and 2 µM *p*-PO_4_^3−^). Prior to assessing the interaction of the elevated pCO_2_ and hyposalinity, the thalli were maintained for 3 days under an irradiance of 60 μmol photon m^−2^ s^−1^ (12 h:12 h light/dark cycle) at 9 °C (close to the temperature in acquisition site), thus allowing samples to recover from excision and equilibrate. The samples were illuminated using cold-white fluorescence lamps (40 W) and the light intensity was determined using an LI-190SA flat quantum sensor (LI-core, NE, USA).

### 4.2. Experimental Design

*S. japonica* were grown at two CO_2_ concentrations (low CO_2_, LC:400 µatm; high CO_2_, HC:1000 µatm) and three salinities (30, 25 and 20 psu) for 4 days. Each treatment was carried out in three 500 mL side-arm flasks, and each flask contained 7 thalli with 500 mL freshly collected and sterilized seawater. The two pCO_2_ levels were achieved using incubators (GXZ-380C-02, Jingnan Instruments Factory, Ningbo, China) by automatically regulating the fluxes of the ambient air only (for 400 µatm) and combining the gases of air and pure CO_2_ (for 1000 µatm). The salinities that were lower than that of the collected seawater (30 psu) were gained by diluting with Milli-Q water. The flasks were placed in incubators with the corresponding pCO_2_ under 60 μmol photon m^−2^ s^−1^ (12 h:12 h light/dark cycle) at 9 °C. In order to avoid nutrient depletion, the thalli were cultured in fresh seawater that was enriched with 25% PESI medium [66]. Before the cultivation, the TA and pH of medium were examined after 24 h of aeration. The pH was measured using a pH meter (Orion STAR A211; Thermo Scientific), and the TA was determined by titration. The carbonate system parameters were calculated using a CO2SYS software [67] based on the known temperature, salinity, TA and pH.

### 4.3. Growth Rates

The fresh weights (FW, g) of sporophytes in each culture were measured to estimate the growth rate. The relative growth rate (RGR; % day^−1^) during the culture period was calculated using Equation (1):

(1) $$\mathrm{RGR}=100\times \left({\mathrm{lnW}}_{t}-{\mathrm{lnW}}_{0}\right)/t\;$$

where W_0_ and W_t_ are the initial and final FW, respectively, and t is the culture period (days).

### 4.4. Chlorophyll Fluorescence

The photosynthetic characteristics of *S. japonica* were assessed by measuring the Chlorophyll (Chl) *a* fluorescence with a Maxi version of an IMAGING-PAM (M-Series, Walz, Effeltrich, Germany). After 10 min of dark adaptation, the relative electron transport rate versus photon flux density (rETR/PFD) curves (rapid light curves, RLC) were constructed with ten stepwise increasing actinic lights (from 0 to 186 μmol photons m^−2^ s^−1^) at 20 s intervals. Six positions on juvenile sporophytes were randomly selected and tested. The maximal rETR (rETR_max_) was obtained from the RLC using the fitting equation [68]:

(2) $$\mathrm{rETR}={\mathrm{rETR}}_{\max}\;\left(1-e^{-\alpha I/{\mathrm{rETR}}_{\max}}\right)$$

where α and E_k_, respectively, represent the initial slope of RLC and photo-saturation irradiance (E_k_ = rETR_max_/α) of *S. japonica*.

The maximum quantum yield (F_v_/F_m_) was directly measured using a saturation pulse (4000 μmol photons m^−2^ s^−1^), and here, F_v_ and F_m_ stand for the variable and the max fluorescence, respectively. Additionally, the non-photochemical quenching (NPQ) was also directly determined after dark adaptation.

### 4.5. Biochemical Compositions

#### 4.5.1. Pigments

Approximately 0.1 g (FW) sample was used for extracting the Chl *a* and carotenoids. The tissue was fully soaked in 10 mL methanol for 24 h in the darkness, and the absorption of the supernatant was measured at 750, 665, 652 and 510 nm using an ultraviolet absorption spectrophotometer (U-2900, HITACHI, Tokyo, Japan). The pigments contents (mg g^−1^ FW) were estimated according to Wellburn [69].

#### 4.5.2. Soluble Carbohydrates

About 0.1 g (FW) tissue was grounded with a small volume of distilled water, and then, the solution was diluted to 10 mL. The homogenate was centrifuged for 5 min at 4000 rpm. 1 mL supernatant was transferred to a 15 mL glass tube, in which 1 mL distilled water and 8 mL anthrone reagent were added. The mixture was bathed in boiled water for 10 min. After cooling it at room temperature, the soluble carbohydrates (SC) content (mg g^−1^ FW) was measured colorimetrically at 620 nm and estimated according to Yemm and Willis [70].

### 4.6. Statistical Analysis

The statistical analysis was performed using IBM SPSS Statistics 25.0 (SPSS Inc., Chicago, IL, USA). Before the parametric tests, the normality (Shapiro–Wilk test) and homogeneity of variance (Levene’s test) of the data were tested. To analyze the effect of salinity (three levels), pCO_2_ (two levels), and their interaction, a two-way ANOVA analysis was performed for all of the parameters. A Tukey’s post hoc test (One-way ANOVA) was also conducted for the multivariate analysis of variance among the different conditions. The significant level was set at *p* < 0.05, *p* < 0.01 or *p* < 0.001. All of the data were shown as the mean ± standard deviation (SD, *n* ≥ 3).

## 5. Conclusions

Although elevated inorganic carbon improves the photosynthesis of photoautotrophs, the elevated pCO_2_ may inhibit the production of the kelp *S. japonica*. Our data suggest that the low salinity exacerbated the OA stress by further reducing the pH and exposing the alga to a more complicated environment. In addition to the growth rate, a further inhibition in correlation to the decreased salinity was also found in the physiological traits, such as the contents of the pigments (Chl *a* and carotenoids) and the photosynthetic rate (rETR_max_). Under the combined conditions of elevated pCO_2_ and decreased salinity, the decreases in both the pigments and F_v_/F_m_ and the increase in the NPQ indicated the photo-protective mechanisms of *S. japonica*. These behaviors, together with the changes in the soluble carbohydrates, may suggest an energetic reallocation in thalli when it was exposed to the stressed factors. This study indicates that future research should focus more on seawater salinity, especially in the areas of coastal farms, to reduce the risks of an elevated pCO_2_ on kelp cultivation.

## Figures and Tables

**Table 1:** Carbonate parameters of seawater at different pCO_2_ levels and salinities. Data are shown as mean value ± SD (*n* = 3). LC and HC depicted the pCO_2_ levels of 400 and 1000 μatm, respectively. The seawater salinities were 30, 25 and 20 (psu). Different letters indicate significant differences (*p* < 0.05) using Tukey’s post hoc test.

Treatment	pCO_2_ (μatm)	pH	HCO_3_^−^ (μmol kg^−1^)	CO_3_^2−^ (μmol kg^−1^)	CO_2_ (μmol kg^−1^)	DIC (μmol kg^−1^)	TA (μmol kg^−1^)
LC-30	400	7.84 ± 0.01 ^f^	2060 ± 13 ^d^	76.5 ± 1.1 ^e^	30.6 ± 0.4 ^a^	2167 ± 14 ^e^	2249 ± 14 ^c^
LC-25		7.80 ± 0.00 ^e^	1798 ± 15 ^b^	52.6 ± 0.4 ^d^	31.5 ± 0.3 ^a^^b^	1882 ± 15 ^c^	1928 ± 15 ^b^
LC-20		7.72 ± 0.03 ^d^	1568 ± 23 ^a^	33.1 ± 1.6 ^b^	35.4 ± 2.4 ^b^	1636 ± 23 ^a^	1650 ± 20 ^a^
HC-30	1000	7.62 ± 0.01 ^c^	2123 ± 10 ^e^	46.7 ± 0.4 ^c^	53.2 ± 0.9 ^c^	2223 ± 10 ^f^	2238 ± 9 ^c^
HC-25		7.55 ± 0.01 ^b^	1857 ± 22 ^c^	30.8 ± 0.5 ^b^	57.4 ± 2.2 ^d^	1945 ± 23 ^d^	1934 ± 20 ^b^
HC-20		7.48 ± 0.01 ^a^	1609 ± 18 ^a^	19.5 ± 0.1 ^a^	63.0 ± 1.4 ^e^	1691 ± 19 ^b^	1657 ± 18 ^a^

## Data Availability

The data of this study are available from the corresponding author upon reasonable request.

## Supplementary Materials

The following supporting information can be downloaded at: https://www.mdpi.com/article/10.3390/plants11212978/s1, Table S1: Two-way ANOVA analyses for the interactive effect of pCO_2_ and salinity on growth, photosynthetic parameters, pigments and soluble carbohydrate contents in *S. japonica* at day 4.
