# Supplementary material for: Decreased Salinity Offsets the Stimulation of Elevated pCO2 on Photosynthesis and Synergistically Inhibits the Growth of Juvenile Sporophyte of Saccharina japonica (Laminariaceae, Phaeophyta)
Source: Plants (Basel). 2022 Nov 4;11(21):2978. doi: 10.3390/plants11212978 (PMC9656199; doi:10.3390/plants11212978)
Supplement: Supplementary file 1 [file plants-11-02978-s001.zip › plants-1977301-supplementary.pdf]

Supplementary data for the article:

Decreased salinity offsets the stimulation of elevated pCO_2 on photosynthesis and synergistically inhibits the growth of juvenile sporophyte of *Saccharina japonica* (Laminariaceae, Phaeophyta)

Table S1. Two-way ANOVA analyses for the interactive effect of pCO_2 and salinity on growth, photosynthetic parameters, pigments and soluble carbohydrate contents in *S. japonica* at day 4.

Factors	df	F	*P*	Partial η^2
**RGR**				
Salinity	2	122.542	0.000	0.953
pCO_2	1	42.147	0.000	0.778
Salinity × pCO_2	2	0.247	0.785	0.040
**Chl *a***				
Salinity	2	8.029	0.006	0.572
pCO_2	1	16.845	0.001	0.584
Salinity × pCO_2	2	1.413	0.218	0.191
**Cartenoid**				
Salinity	2	11.686	0.002	0.661
pCO_2	1	17.752	0.001	0.597
Salinity × pCO_2	2	3.911	0.049	0.395
**F_v/F_m**				
Salinity	2	20.141	0.000	0.573
pCO_2	1	3.024	0.092	0.092
Salinity × pCO_2	2	0.259	0.774	0.017
**$rETR_{max}$**				
Salinity	2	1.990	0.154	0.117
pCO_2	1	14.479	0.001	0.326
Salinity × pCO_2	2	7.236	0.003	0.325
**E_k**				
Salinity	2	3.168	0.056	0.174
pCO_2	1	6.703	0.015	0.183
Salinity × pCO_2	2	3.904	0.031	0.207
**α**				
Salinity	2	6.006	0.006	0.286
pCO_2	1	0.004	0.950	0.000
Salinity × pCO_2	2	4.714	0.017	0.239
**NPQ**				
Salinity	2	0.581	0.566	0.041
pCO_2	1	1.350	0.255	0.048
Salinity × pCO_2	2	4.782	0.017	0.262
**Soluble carbohydrates**				
Salinity	2	48.210	0.000	0.889
pCO_2	1	29.893	0.000	0.714
Salinity × pCO_2	2	7.651	0.007	0.560
